# Recognition and Explainable Quantitative Evaluation of Fundamental Taekwondo Kicks from Smartphone Videos

**DOI:** 10.3390/s26144499

**Published:** 2026-07-15

**Authors:** Zenan Wang, Shuo Sun, Xilin Liang, Linhua Chen

**Affiliations:** 1 College of Sports Science, Dankook University, Cheonan Campus, Cheonan 31116, Republic of Korea; wangzenan436@gmail.com; 2 School of Physical Education, Huzhou University, Huzhou 313000, China; 3 School of Humanities, Zhejiang Business College, Hangzhou 310053, China

**Keywords:** smartphone video, pose estimation, action recognition, action quality assessment, taekwondo, explainable feedback

## Abstract

Accessible and quantitative assessment of rapid martial arts movements remains difficult without specialized equipment or continuous expert observation. Here, we present a desktop prototype that uses smartphone-recorded videos to recognize and evaluate three fundamental taekwondo kicks: front, roundhouse and axe kicks. The TKD-Kick3 protocol planned 790 recordings from 150 university students across five physical education classes and retained 765 cleaned pose sequences; because two first-year classes had not yet learned the roundhouse kick, front and axe kick recordings were planned for all students, whereas roundhouse kick recordings were planned only for 95 second-year students. MediaPipe Pose extracted 13 body keypoints, which were encoded as 52-dimensional frame features combining normalized two-dimensional positions and first-order velocities. Because participant identifiers were unavailable, recognition was assessed using a file-level validation split; recordings from the same participant may therefore have crossed subsets, potentially inflating performance estimates. Across three random seeds, the selected BiLSTM with uniform resampling and argmax inference achieved 75.3 ± 2.3% accuracy, 74.3 ± 2.3% macro-F1 and 74.5 ± 2.3% balanced accuracy; within the same validation setting, the best Transformer configuration achieved 67.3 ± 1.7% accuracy. On 30 expert-annotated videos, system scores showed moderate association with expert ratings (Spearman’s rho = 0.627; mean absolute error = 0.601 on a 10-point scale), providing preliminary support for the interpretable scoring approach. These results provide proof-of-concept evidence for smartphone-based kick assessment but do not establish participant-independent generalization or expert-equivalent scoring, motivating future evaluation with participant-level metadata and independent test cohorts.

## 1. Introduction

### 1.1. Motivation and Significance

With the rapid development of mobile intelligent terminals and AI-assisted perception, sports teaching and training are increasingly transitioning from experience-driven practice to data-driven and feedback-centric paradigms. In taekwondo fundamentals training, traditional classes typically require an instructor to demonstrate, organize practice, and correct technique for 30–40 students within limited time. Under such conditions, instructors often provide only sparse verbal feedback at a few critical moments, making it difficult to build process-level evidence that can be replayed, compared, and reviewed. Moreover, assessment criteria may vary with the instructor’s experience, attention load, and classroom constraints, leading to inconsistent training targets and limited reproducibility.

Advances in human pose estimation and skeleton-based action recognition enable automatic extraction of joint trajectories from videos and support downstream behavior modeling [1,2]. Lightweight pipelines such as MediaPipe and BlazePose can also support on-device keypoint tracking [3,4]. A key research question is how lightweight pose estimation, temporal modeling [5], transparent scoring rules [6], and phase-aware temporal normalization [7] can be integrated into a video-based assessment pipeline that accepts smartphone recordings from typical educational environments. The present study evaluates the technical feasibility of this integration; its potential value for classroom support and self-guided practice requires future educational validation.

This study targets fundamental taekwondo kick training and develops an end-to-end pipeline comprising smartphone video capture, keypoint extraction, action recognition, quantitative scoring, and visual feedback. From a methodological perspective, we examine the mapping between spatiotemporal skeletal features and transparent scoring criteria. From an application perspective, the prototype is designed to generate itemized and visualized correction cues. Its effectiveness for classroom instruction or at-home learning was not evaluated in the present study.

### 1.2. Limitations of Traditional Assessment and Existing Solutions

Traditional teaching assessment is often coarse-grained (e.g., pass/fail or broad grade levels) and rarely decomposes performance into technique-relevant factors such as supporting leg stability, kicking height, or landing position. Students thus struggle to understand where the error occurs and how to correct it. Due to limited time and attention resources, instructors cannot provide frame-level analysis for every student, and assessment lacks objective, replayable evidence.

While deep-learning-based pose estimation is widely used, directly deploying it for taekwondo instruction still faces several challenges: (1) many studies emphasize classification accuracy but do not provide scoring mechanisms aligned with instructional criteria (e.g., accuracy and expressiveness); (2) some systems rely on specialized or costly equipment, limiting adoption; and (3) for fast kicking actions, general-purpose models can be sensitive to short-term keypoint dropout and jitter, which degrades both recognition and metric extraction.

### 1.3. Method Overview and Contributions

To address subjective assessment, delayed feedback, and the difficulty of producing quantified and traceable evaluations in fundamental taekwondo training, we developed a video-based assessment pipeline using smartphone recordings. The specific contributions are threefold: (1) an efficient 13-keypoint, 52-dimensional representation with visibility-informed preprocessing for short kicking sequences; (2) a controlled investigation of pivot-based phase-aligned resampling and deployable class hypothesis sweep inference across BiLSTM, Transformer, and GCN backbones; and (3) integration of three-class recognition with a transparent 10-point rule-based scoring scheme that produces itemized deduction reasons and visual feedback. These are system-level and application-oriented contributions; MediaPipe Pose, BiLSTM, Transformer, GCN, and rule-based scoring are established techniques and are not claimed as individually novel. The current system is a CPU-based desktop implementation that analyzes smartphone-recorded videos; on-device mobile deployment was not evaluated.

The system takes smartphone recordings as its only input. We use MediaPipe Pose to extract 13 skeletal keypoints and retain per-keypoint visibility to handle occlusion and dropout [3]. To reduce variations due to body shape and camera placement, we perform per-frame translation removal and scale normalization. The center is prioritized as the midpoint of left/right hips; if hips are unreliable, we fall back to the midpoint of shoulders; otherwise, we use the mean of all visible points. The scale factor is the maximum of shoulder width and hip width with a lower bound. The normalized two-dimensional coordinates and first-order velocity are concatenated to form a 52-dimensional feature per frame (D = 52).

To mitigate phase drift caused by different motion tempos, we introduce a pivot-based phase-aligned resampling strategy (Phase-Aligned Resize, PA) that standardizes sequences to T = 96. For front and roundhouse kicks, the pivot is located using the peak distance between the attacking ankle and ipsilateral hip; for axe kicks, the pivot is the frame at which the attacking ankle reaches maximal height. A class-dependent non-symmetric window is extracted around the pivot and resampled to align key phases (chamber, strike, recovery, and landing) across samples. Because the alignment rule depends on the class, but the class is unknown at inference, PA-Sweep evaluates one class-conditioned alignment candidate per target class. For candidate class c, the sequence is aligned using the pivot and window defined for c, passed through the recognition model, and scored by the posterior probability assigned to the same class c. The predicted class is the candidate for which this class-matched posterior probability is highest.

For recognition, we evaluate three temporal backbones: a lightweight Transformer encoder [5], a bidirectional LSTM [8], and a GCN-style baseline [9,10]. The final backbone was selected according to the reproduced validation results rather than being predefined. In the current experiment matrix, the BiLSTM with uniform temporal resampling and argmax inference provides the strongest recognition performance.

For quality evaluation, we prioritize interpretability. Inspired by WT poomsae scoring, we design a 10-point scoring rubric decomposed into accuracy (4 points) and expressiveness (6 points) [6]. Instead of black-box regression, we compute kinematic metrics to generate itemized scores and explicit deduction reasons (e.g., supporting leg stability, kicking height and knee extension, chamber amplitude, recovery/axe height, landing position, and hand stability). The system outputs a feedback video, overlaying skeletons, sub-scores, and diagnostic messages, with an adaptive layout that switches between side-panel and bottom-panel modes based on the video aspect ratio.

## 2. Related Work

### 2.1. Transformer Models for Skeleton-Based Action Recognition

Transformer architectures, empowered by multi-head self-attention, provide strong capability for modeling long-range dependencies and parallel computation, and were first introduced in natural language processing [5]. They have since been adopted for vision tasks by representing images or videos as sequences of tokens, enabling efficient global aggregation [11].

In skeleton-based action recognition, graph convolutional baselines such as ST-GCN [9] model human topology and have been widely used. Transformers have become a prominent paradigm in this area. Prior work embeds joint coordinates as token sequences and uses self-attention to model spatial dependencies across joints and temporal patterns over long horizons, alleviating the limited receptive field of convolutional networks [12,13]. Hybrid GCN-Transformer designs have also been explored [14]. Despite their generality, Transformers may face optimization challenges in small-data scenarios, motivating lightweight adaptations for specific actions (e.g., taekwondo kicks) and task-specific temporal normalization.

### 2.2. Action Quality Assessment in Sports

Action quality assessment (AQA) aims to quantify the degree of standardization and performance quality, and is central to intelligent sports instruction.

Early approaches used template matching, such as Dynamic Time Warping (DTW), to measure distances between a learner’s skeleton sequence and an expert template. While simple, such methods are sensitive to tempo differences and heavily depend on the quality of expert templates.

With the rise of deep learning, regression-based methods have become mainstream, extracting video features using 3D-CNNs or temporal networks and directly regressing a scalar score [15]. These methods can achieve strong performance on benchmarks but often lack interpretability, limiting their pedagogical utility.

Rule-based kinematic evaluation provides an alternative by computing physical parameters (e.g., joint angles, angular velocities, center-of-mass trajectories) and comparing them against instructional criteria [16]. Temporal segment graph convolutional networks have also been proposed to model temporal structure in skeleton-based action recognition [17]. This work adopts the rule-based philosophy and tailors metrics and scoring items to taekwondo fundamentals while ensuring robustness to keypoint noise.

## 3. Methods

### 3.1. System Overview

**Figure 1:**
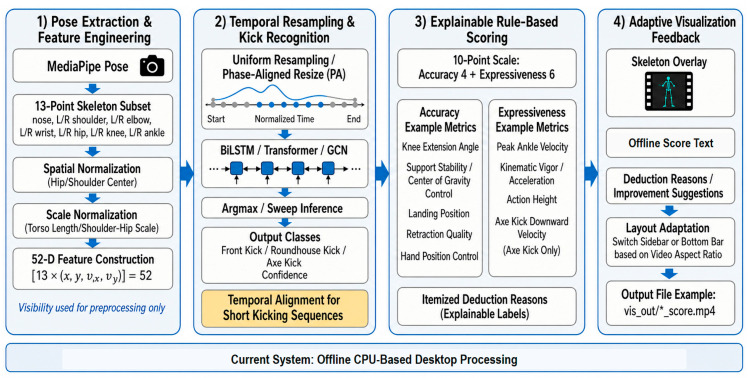
Overall pipeline of the video-based taekwondo kick recognition and scoring prototype using smartphone recordings. Arrows indicate the processing flow, different colors distinguish the main functional modules, and the asterisk (*) indicates the video file name.

We propose a video-based framework using smartphone recordings to recognize and quantitatively evaluate fundamental taekwondo kicks. As illustrated in Figure 1, the system integrates four core modules: skeletal data acquisition and feature engineering, temporal recognition, rule-based scoring, and visual feedback generation. The current prototype processes smartphone-recorded videos on a desktop CPU.

Given a smartphone recording, the system identifies one primary target according to the largest motion extent in the scene and performs pose-based analysis only on that target. MediaPipe Pose then extracts a 13-keypoint trajectory for the selected subject, covering the head (with the nose used as a proxy), shoulders, elbows, wrists, hips, knees, and ankles, together with per-keypoint visibility values [3]. The visibility values were retained for preprocessing and reliability checks but were not concatenated into the recognition model input. We then apply translation removal and scale normalization. The recognition module in the final prototype uses a BiLSTM model with uniform temporal resampling and argmax inference for three-class classification, including front kick, roundhouse kick, and axe kick. Transformer- and GCN-based models were retained as comparison backbones. The scoring module follows an interpretable scheme inspired by WT scoring [6]. The feedback module overlays skeletons, sub-scores, and textual suggestions and adaptively chooses a side-panel or bottom-panel layout according to the video aspect ratio.

### 3.2. Skeletal Feature Engineering and Temporal Resampling

#### 3.2.1. Spatial Normalization

Let the raw skeleton sequence be S ∈ R^(T_raw×J×2)^, where T_raw is the number of frames and J = 13 is the number of keypoints. To improve robustness against occlusion and dropout, we prioritize the midpoint of left/right hips as the translation center; if hips are unreliable, we fall back to the midpoint of shoulders; otherwise, we use the mean over all keypoints [18]. We define a visibility threshold τ (e.g., τ = 0.5) to determine whether hips or shoulders are available: a body part is considered available when both left and right keypoints have visibility greater than τ.

(1) $$C_{t}=\left\{\begin{array}{ll} \frac{P_{t,\text{L\_Hip}}+P_{t,\text{R\_Hip}}}{2}, & \mathrm{hips}\;\mathrm{available}\\ \frac{P_{t,\text{L\_Shoulder}}+P_{t,\text{R\_Shoulder}}}{2}, & \mathrm{shoulders}\;\mathrm{available}\\ \frac{1}{J}\sum_{j=1}^{J}P_{t,j}, & \mathrm{otherwise} \end{array}\right.$$

We set the scale factor as the maximum of shoulder width and hip width with a lower bound of 1.0 to avoid numerical instability:

(2) $$L_{t}=max\left(∥P_{t,\text{L\_Shoulder}}-P_{t,\text{R\_Shoulder}}{∥}_{2},\;∥P_{t,\text{L\_Hip}}-P_{t,\text{R\_Hip}}{∥}_{2},1.0\right)$$

All keypoints are centered and normalized to obtain normalized 2D coordinates:

(3) $${\hat{P}}_{t,j}=\frac{P_{t,j}-C_{t}}{L_{t}}$$

#### 3.2.2. High-Dimensional Feature Construction

After temporal resampling, all sequences are standardized to *T*_target_ = 96 (denoted as *T*). For frame *t*, we define the feature vector:

(4) $$f_{t}=\mathrm{Concat}\left(F_{\mathrm{pos}}\left(t\right),F_{\mathrm{vel}}\left(t\right)\right)\in R^{52},$$

where *F*_pos_(*t*) is the concatenation of 13 normalized 2D keypoints (26D), and *F*_vel_(*t*) = $\hat{P_{t}}-{\hat{P}}_{t-1}$ is the first-order velocity (26D), with *F*_vel_ (1) = 0. Visibility values were not included in the 52-dimensional feature vector. They were used only for preprocessing decisions, including keypoint availability checks, fallback selection of the normalization center, and handling of low-confidence detections.

#### 3.2.3. Pivot-Based Phase-Aligned Resize (PA)

Different students exhibit substantial tempo variation; naive linear interpolation may cause key strike moments to drift along the temporal axis. We therefore adopt a pivot-based phase-aligned resampling strategy, drawing on the broader idea that phase-aware temporal normalization can reduce timing variation in sequence comparison [7].

Pivot definition and localization. During training and offline analysis, the action class is known, and we apply class-specific pivot rules. For front and roundhouse kicks, the pivot is the frame where the distance ||*P*_t,ank_ − *P*_t,hip_||2 between the attacking ankle and ipsilateral hip is maximized. For axe kicks, the pivot is the frame where the attacking ankle reaches the maximum height (minimum *y* in image coordinates).

During online inference, the class is unknown. To deploy class-dependent alignment without relying on ground truth, we use a sweep strategy: we run alignment and a forward pass for each candidate class and select the class with the highest predicted probability under its corresponding candidate alignment.

Non-symmetric windowing and resampling. Given pivot frame *t*_pivot_, we extract a non-symmetric temporal window:

(5) $$w_{s}=\left\lfloor 0.45\;T_{raw}\right\rfloor ,\;w_{e}=\left\lfloor 0.55\;T_{raw}\right\rfloor ,$$

with window boundaries:

(6) $$\left[t_{s},t_{e}\right]=\left[\max\left(1,\;t_{pivot}-w_{s}\right),\;\min\left(T_{raw},\;t_{pivot}+w_{e}\right)\right].$$

We then linearly interpolate within [*t*_s_, *t*_e_] to obtain a fixed-length sequence with *T*_target_ = 96.

### 3.3. Recognition Backbones and Final Model Selection

We evaluated three temporal recognition backbones for three-class kick classification: a bidirectional long short-term memory network (BiLSTM), a lightweight Transformer encoder, and a GCN-style skeleton baseline. All models used the same 96-frame input sequence and the same 52-dimensional frame-level feature representation, consisting of normalized two-dimensional keypoint coordinates and first-order velocities.

The final recognition backbone was selected according to the validation results rather than being predefined. Under the current sample-level validation protocol, the BiLSTM with uniform temporal resampling and argmax inference achieved the highest overall performance and was therefore used as the recognition module in the final prototype.

The selected BiLSTM model consisted of two bidirectional recurrent layers with a hidden size of 128 and dropout of 0.2. The model encoded the temporal evolution of the pose sequence in both forward and backward directions and produced class probabilities for front kick, roundhouse kick, and axe kick through a fully connected classification layer followed by Softmax normalization.

For comparison, we also implemented a lightweight Transformer encoder with a 128-dimensional latent representation, two encoder layers, four attention heads, a feed-forward dimension of 256, and dropout of 0.2. The Transformer was evaluated under both uniform temporal resampling and phase-aligned resampling with sweep inference. In addition, a GCN-style baseline was included to examine the performance of a graph-based skeleton representation under the same input and validation setting.

This design allowed the recognition experiment to compare recurrent, attention-based, and graph-based temporal modeling strategies while keeping the input feature construction and validation protocol consistent across models.

### 3.4. Explainable Scoring Rules

To avoid the “black-box regression” limitation and provide instructive feedback, we adopt an interpretable, kinematics-driven scoring framework. The total score is 10 points, decomposed into accuracy *S*_acc_ (max 4) and expressiveness *S*_expr_ (max 6):

(7) $$S_{total}=S_{acc}+S_{expr},\;S_{acc}\in \left[0,4\right],\;S_{expr}\in \left[0,6\right].$$

Inputs include keypoints and visibility (kpts,vis) and video frame rate fps. We normalize keypoints as described in Section 3.2, compute robust kinematic metrics using EMA smoothing and quantile statistics (RawMetrics), and map them to scores and deduction reasons.

#### 3.4.1. Metric Extraction (Raw Metrics)

Key metrics included peak attacking ankle speed, action height, temporal smoothness, and a pose-derived kinematic vigor index. For axe kicks, downward ankle velocity was additionally computed to characterize the downward-striking phase. Accuracy-related metrics included the median supporting knee angle, normalized kick height relative to the hip, peak kick-knee extension, roundhouse alignment deviation, chamber amplitude, recovery ratio, forward landing displacement, and hand stability.

#### 3.4.2. Accuracy Score (4 Points Total)

Accuracy includes six items (weights sum to 4.0):

- Supporting leg extension (0.5): mapped from median supporting knee angle *θ*_med_^sup^, where 150° yields 0 and 170° approaches full credit.
- Kicking technique (1.8): composed of height attainment (60%) and knee extension (40%). Knee extension is linearly mapped from *θ*_max_^kick^ in 150–180°. For roundhouse kicks, an additional alignment penalty (up to 0.4) constrains shoulder–hip–knee axis consistency.
- Chamber (1.0): based on vertical knee amplitude; 0.15 approaches full credit.
- Recovery/axe height (0.3): for non-axe kicks, combines recovery ratio and post-peak re-bending amplitude; for axe kicks, uses normalized ankle peak height in the “chest-to-head” range.
- Forward landing (0.2): checks whether the attacking ankle lands in front of the supporting ankle along the body forward axis.
- Hand position stability (0.2): based on the 95th percentile displacement of wrists relative to the initial baseline; ≤0.10 approaches full credit and ≥0.35 yields 0.

#### 3.4.3. Expressiveness Score (6 Points Total)

Expressiveness was computed from pose-derived speed, action height, smoothness, and kinematic vigor, with the component weights summing to 6.0. For axe kicks, downward ankle velocity was additionally considered to reflect the downward-striking phase. Raw metrics were mapped to normalized component scores in the range [0, 1] using predefined score ranges and then combined according to the scoring weights. Because all expressiveness metrics were derived from two-dimensional monocular pose trajectories, the kinematic vigor index should not be interpreted as biomechanical power.

To avoid unrealistically low expressiveness when accuracy is high, we use a mild coupling floor:

(8) $$S_{expr}\leftarrow max\left(S_{expr},\;min\left(6,\;1.0S_{acc}+0.5\right)\right).\;$$

#### 3.4.4. Diagnosis and Feedback Generation

The system ranks accuracy deductions and outputs the most significant deduction items with corresponding reasons in the feedback panel. Skeleton overlays and computed scores are composited into the output video.

## 4. Experiments and Analysis

### 4.1. Dataset and Current Validation Protocol

The TKD-Kick3 acquisition protocol included 150 university students from five routine physical education classes: two first-year classes and three second-year classes. The protocol was not fully balanced across kick types because the two first-year classes had not yet learned the roundhouse kick at the time of recording. Therefore, all students were asked to record two front-kick videos and two axe kick videos, whereas only the 95 students from the three second-year classes were asked to record two roundhouse kick videos. This resulted in 790 planned smartphone-recorded videos at the protocol level, including 300 front-kick videos, 190 roundhouse kick videos, and 300 axe kick videos. After data screening, the active repository contained 765 retained three-class pose sequences. After data screening, the active repository contained 765 retained three-class pose sequences. The acquisition protocol is summarized in Table 1.

A critical audit found that participant identifiers were not available in the active sequence files or in a separate metadata table. Therefore, a participant-independent split could not be generated from the current repository. The current experiments used the existing cleaned file-level training and validation split and should be interpreted as sample-level validation rather than participant-independent testing. Because multiple videos were recorded for each participant, videos from the same participant may appear in both subsets. This potential participant overlap can introduce information leakage and yield optimistic validation estimates, thereby limiting evidence of generalization to unseen participants. The dataset inclusion, exclusion, and current split summary are presented in Table 2.

### 4.2. Model Configurations and Training

All recognition models used 96-frame sequences with 52-dimensional frame features. Training was repeated with seeds 0, 1, and 2. Adam was used with an initial learning rate of 1 × 10^−3^, batch size 8, dropout 0.2, and 40 epochs. To reduce class-frequency imbalance during training, all three backbones used inverse-frequency sampling through a class-balanced WeightedRandomSampler. The Transformer additionally used inverse-frequency class weights in the cross-entropy loss, label smoothing of 0.05, and training-only temporal jitter; the LSTM and GCN baseline scripts used unweighted cross-entropy and no label smoothing. No additional spatial or synthetic-pose augmentation was used in the reported experiments. The model architectures and training configurations are summarized in Table 3.

### 4.3. Recognition Results

The strongest recognition result was obtained by the BiLSTM with uniform temporal resampling and argmax inference. This configuration achieved 75.3 ± 2.3% accuracy, 74.3 ± 2.3% macro-F1, and 74.5 ± 2.3% balanced accuracy across three seeds. LSTM-PA-Sweep was also run and reached 71.2 ± 1.0% accuracy, so phase-aligned sweep inference did not improve the LSTM under the current split. Here, UNI-Argmax denotes uniform temporal resampling followed by standard argmax inference over the predicted class probabilities. The recognition performance of the three backbones and configurations is summarized in Table 4.

The Transformer with combined phase-aligned resampling and sweep inference reached 67.3 ± 1.7% accuracy, compared with 62.2 ± 2.4% for Transformer-UNI-Argmax. The present results identify LSTM-UNI as the best-performing recognition backbone in the current experiment matrix. Because each configuration was repeated over only three random seeds, the study did not treat seed-level variation as sufficient for robust formal significance testing. Model comparisons were therefore interpreted descriptively using mean ± standard deviation across seeds.

Table 5 further separates the effect of temporal normalization and inference strategy in the Transformer model. UNI-Argmax removes phase-aligned resampling and uses uniform temporal resampling as the baseline. PA-SingleHyp. introduces phase-aligned resampling but does not use the full class hypothesis sweep. PA-Sweep combines class-conditioned phase-aligned resampling with sweep inference and produced the best deployable Transformer result. PA-Oracle uses the ground-truth class for alignment and is reported only as a non-deployable analysis upper bound. The gradual improvement from UNI-Argmax to PA-Sweep suggests that temporal alignment and class-conditioned inference were beneficial for the Transformer, although the remaining gap between PA-Sweep and PA-Oracle indicates that imperfect class hypothesis selection still limits performance.

The class-wise performance of the selected LSTM-UNI configuration is presented in Table 6. These results indicate that the selected LSTM-UNI configuration provided the strongest recognition performance within the current file-level validation protocol; participant-independent generalization is addressed separately as a dataset limitation in Section 4.1 and Section 5.3.

The retained dataset remained imbalanced, with fewer roundhouse kick sequences than front-kick or axe kick sequences (190 vs. 288 and 287). In the validation subset, the roundhouse kick also had the lowest support (25 vs. 38 for front kick and 41 for axe kick) and the lowest class-wise F1 score (67.9% vs. 73.9% and 81.0%). This pattern may reflect the combined influence of reduced class coverage, cohort/training–experience imbalance, and the kinematic complexity of the roundhouse kick.

Compared with front and axe kicks, which are dominated by more linear sagittal or vertical trajectories, the roundhouse kick contains a stronger rotational and transverse-plane component. This movement pattern can increase viewpoint sensitivity and self-occlusion of the hip, knee, and ankle joints, especially when the attacking leg rotates across the body or is partially hidden by the trunk. Such monocular tracking noise may reduce the stability of the extracted two-dimensional keypoint trajectories and make the roundhouse class harder to separate from the other kicks. Therefore, the lower roundhouse performance should not be attributed to sample size alone.

**Figure 2:**
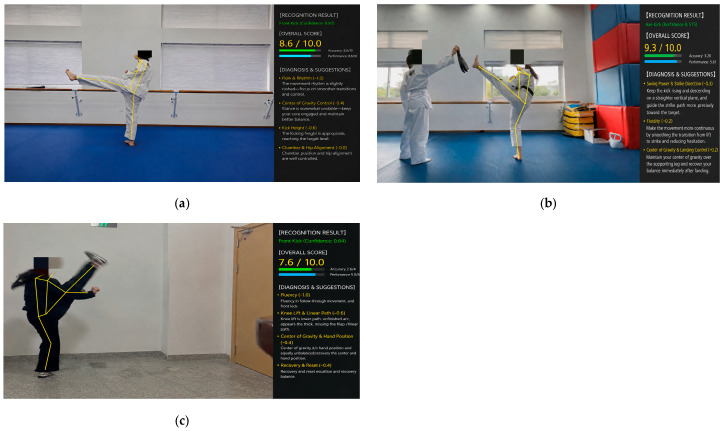
Examples of anonymized visual feedback generated by the proposed system in the human–AI agreement subset: (**a**) front kick; (**b**) axe kick; and (**c**) front-kick example with a lower system score. Identifying facial and background information was removed or obscured for publication.

**Figure 3:**
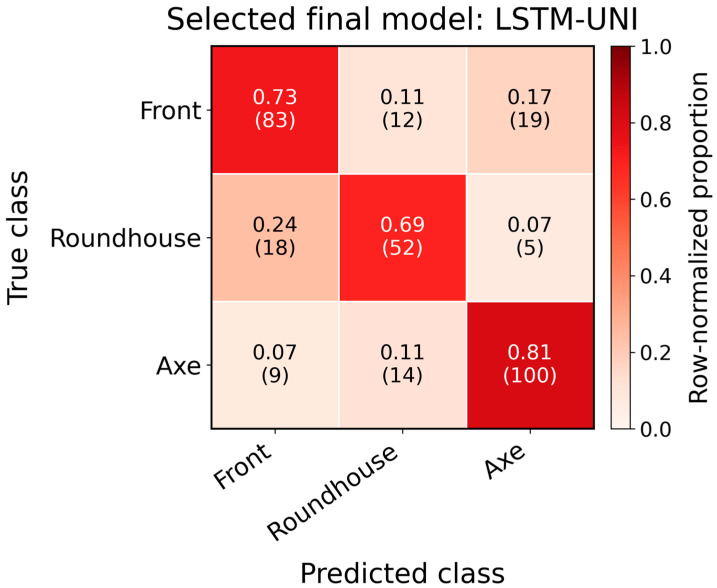
Row-normalized confusion matrix of the selected LSTM-UNI recognition model.

We report macro-F1 and balanced accuracy in addition to overall accuracy to reduce the influence of class-frequency bias. Class-balanced sampling mitigated unequal training exposure, but it cannot remove acquisition bias, cohort confounding, or the need for a balanced participant-level dataset. Representative examples of the visual feedback generated by the proposed system are shown in Figure 2. The row-normalized confusion matrix of the selected LSTM-UNI model is presented in Figure 3.

### 4.4. Runtime Performance

Runtime was measured using the current CPU-based desktop implementation on 30 validation videos, with 10 videos per class. The measurements were performed on a desktop computer with an Intel(R) Core (TM) i5-10400F CPU @ 2.90GHz, 31.9 GB RAM, and Microsoft Windows 10 Pro 64-bit (version 10.0.19045). No GPU acceleration was used for the reported runtime measurements. The total processing time excluding feedback rendering was 7.53 ± 4.53 s per video. Recognition inference itself required only 0.0030 ± 0.0014 s per video; pose extraction was the dominant computational stage. These results describe offline desktop processing and depend on video duration, hardware, software implementation, and the pose-estimation backend. They should not be interpreted as evidence of real-time or on-device smartphone inference. The detailed runtime results are summarized in Table 7.

### 4.5. Explainable Scoring and Human–AI Agreement

The scoring module produced a 10-point score consisting of 4 points for accuracy and 6 points for expressiveness. The expressiveness component was computed from pose-derived speed, action height, smoothness, and a kinematic vigor index. For axe kicks, downward ankle velocity was additionally included. The kinematic vigor index should not be interpreted as biomechanical power because no force, torque, or three-dimensional dynamics were measured. Expert-score reliability was assessed using the intraclass correlation coefficient [19], class agreement was evaluated using Cohen’s kappa [20], and score agreement was further examined using Bland–Altman analysis [21]. The human–AI agreement results are summarized in Table 8.

**Figure 4:**
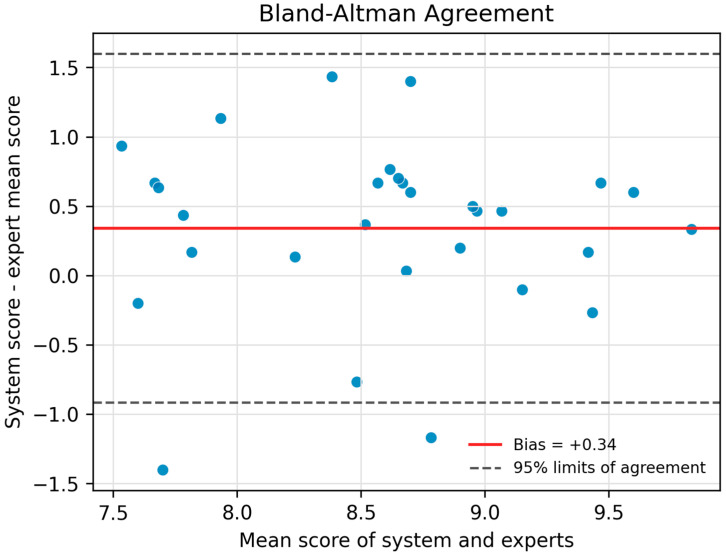
Bland–Altman plot comparing system scores with mean expert scores. Each blue dot represents one expert-annotated video.

The Bland–Altman agreement between the system scores and mean expert scores is illustrated in Figure 4. These results provide preliminary evidence that the rule-based scoring layer is directionally consistent with expert evaluation. However, the analysis included only 30 expert-annotated videos, and the subset cannot currently be linked to a confirmed participant-independent test set. The estimates are therefore exploratory and should not be generalized to broader populations, recording conditions, or expert pane.

## 5. Discussion

### 5.1. Recognition Backbone and Temporal Alignment

The reproduced experiment matrix showed that the BiLSTM with uniform temporal resampling and argmax inference provided the strongest recognition performance under the current sample-level validation protocol. Although the Transformer benefited from the combined phase-aligned resampling and sweep-inference configuration, its best performance remained lower than that of the BiLSTM. The final prototype therefore used the LSTM-UNI configuration as the recognition backbone, while the Transformer and GCN models were interpreted as comparison backbones.

The different effects of PA-Sweep across backbones may be related to their temporal modeling mechanisms. The Transformer may benefit from phase-aligned resampling because self-attention compares information across temporal positions, and aligning key phases such as chamber, strike, recovery, and landing can make informative temporal tokens more comparable across samples. In contrast, the LSTM processes sequences recurrently and may already capture local temporal transitions and velocity patterns under uniform resampling.

Moreover, PA-Sweep applies class-dependent temporal windows during inference. When the candidate alignment does not match the true action class, the resulting crop may introduce phase noise or partial boundary truncation. PA-Sweep also requires one alignment and forward pass per candidate class, increasing inference cost relative to uniform resampling. Its practical advantage is therefore architecture-dependent: it improved the Transformer in the controlled comparison but did not improve the BiLSTM, for which uniform resampling was retained in the final prototype. PA-Sweep should be viewed as an optional normalization strategy rather than a universally superior replacement for uniform resampling. Taken together, PA-Sweep had different effects across the three backbones: it improved the Transformer by making key temporal phases more comparable, did not improve the BiLSTM because the recurrent model already captured local temporal transitions under uniform resampling, and provided little benefit for the GCN baseline, the simplified graph representation of which showed limited capacity under the current small and imbalanced dataset.

### 5.2. Explainable Scoring and Expert Agreement

The rule-based scoring module provides interpretable feedback aligned with expert-relevant action features such as kicking height, knee extension, recovery, stability, speed, and smoothness. However, all metrics are derived from two-dimensional monocular pose trajectories. The kinematic vigor index should not be interpreted as true biomechanical power.

Previously reported Taekwondo systems address related but not directly equivalent tasks. Hwang et al. [15] focused on classifying Poomsae side-kick performance using kinematic parameters and physical characteristics, whereas Fernando et al. [16] used MediaPipe-derived skeleton points and an LSTM to evaluate Poomsae movements, reporting 61% accuracy against domain expert results. The present study considers three kick categories and combines recognition with itemized rule-based scoring and visual feedback from smartphone recordings. However, differences in movement scope, labels, datasets, validation protocols, and outcome definitions make direct numerical ranking inappropriate. In particular, the current file-level split and small expert subset do not support claims of superior generalization over these systems.

As shown in Table 9, the present framework is not intended to replace prior Taekwondo assessment systems or to claim direct numerical superiority. Rather, it complements existing work by combining smartphone-recorded video input, three-class fundamental kick recognition, transparent rule-based scoring, itemized deduction reasons, and visual feedback generation within a single feasibility-oriented prototype.

In this study, explainability refers to intrinsic rule transparency in the scoring module: each sub-score and deduction can be traced to a named kinematic metric, threshold, and scoring rule. This differs from post hoc explainable artificial intelligence methods, such as saliency or feature-attribution analyses applied to a black-box recognition model. The BiLSTM recognition backbone is not itself interpreted by an XAI method; rather, recognition and the transparent rule-based scoring layer are separate components. This design improves traceability of feedback but depends on manually specified criteria and may require recalibration for other populations, techniques, or recording conditions.

The expert-annotated subset showed encouraging but preliminary agreement, including high inter-expert score reliability and moderate system-expert score association. Nevertheless, the analysis contains only 30 videos and cannot currently be verified as independent at the participant level. The limited subset constrains the precision and generalizability of the agreement estimates. These results support feasibility only and do not establish expert-equivalent scoring.

### 5.3. Limitations and Future Work

The primary limitation of the present dataset is the absence of participant identifiers in the active sequence files. As a result, the current evaluation could not enforce participant-level separation between training and validation samples. Because each participant contributed multiple recordings, the same participant may be represented in both subsets, allowing subject-specific movement patterns to be shared across training and validation data. This potential leakage may lead to optimistic recognition estimates. The reported results therefore reflect sample-level validation under the existing file-level split and should not be interpreted as evidence of participant-independent generalization. Future work should recover or reconstruct participant-level metadata and repeat the full experiment matrix using subject-independent train, validation, and test partitions.

Another limitation is the class and cohort imbalance created by the acquisition protocol. Because first-year students had not yet learned the roundhouse kick, roundhouse kick samples were collected only from second-year students and were fewer in number, whereas front-kick and axe kick samples included both first- and second-year students. The classifier may therefore capture differences in training experience, movement stability, or class-specific execution patterns in addition to the intended action-category differences. Class-balanced sampling reduced unequal exposure during model training, but it did not remove the underlying cohort confounding, and the minority roundhouse class still showed the lowest validation support and F1 score. Future data collection should balance all target kick types across participants, year levels, and skill backgrounds and evaluate augmentation strategies within a participant-independent protocol.

Additional limitations include monocular two-dimensional sensing, sensitivity to occlusion and camera viewpoint, possible multi-person interference, classroom-specific recording conditions, and the absence of on-device smartphone runtime testing. The study provides an initial validation of the recognition and scoring workflow; it does not demonstrate improved learning outcomes. Accordingly, immediate instructional benefit, scalable classroom use, and self-guided practice should be regarded as potential future applications that require prospective educational and deployment studies.

## 6. Conclusions

This study developed and audited a video-based desktop prototype that uses smartphone recordings to recognize and quantitatively evaluate three fundamental taekwondo kicks: front kick, roundhouse kick, and axe kick. The system used MediaPipe Pose to extract 13 body keypoints from monocular recordings and constructed 52-dimensional frame-level features from normalized two-dimensional coordinates and first-order velocities. On this basis, the prototype integrated temporal kick recognition, transparent rule-based scoring, and visual feedback generation.

Under the current sample-level validation protocol, the selected recognition backbone was the BiLSTM with uniform temporal resampling and argmax inference. This configuration achieved 75.3 ± 2.3% accuracy, 74.3 ± 2.3% macro-F1, and 74.5 ± 2.3% balanced accuracy across three random seeds. Transformer- and GCN-based models were retained as comparison backbones, with the best Transformer configuration reaching 67.3 ± 1.7% accuracy. These results indicate that the recurrent model provided the most stable recognition performance in the present experimental setting, although they should not be interpreted as evidence of general superiority over Transformer-based approaches.

The expert-annotated subset provided preliminary evidence that the rule-based scoring module was directionally consistent with human evaluation. However, the subset was limited to 30 videos, and the scoring metrics were derived from two-dimensional monocular pose trajectories. Therefore, the present results do not support claims of expert-equivalent judging, biomechanical power estimation, or deployment-ready automated assessment.

The main limitations of the current study are the absence of participant identifiers in the active sequence files and the unbalanced acquisition protocol across kick types. The former prevented participant-level separation between training and validation samples, whereas the latter may have introduced potential cohort- and training–experience bias because roundhouse kick samples were collected only from second-year students. Consequently, the reported recognition results should be interpreted as sample-level validation rather than participant-independent testing. Future work should recover or reconstruct participant-level metadata, balance all target kick types across participants and skill levels, and repeat the full experiment matrix using participant-level train, validation, and test partitions. Further validation should also include additional recording environments, camera viewpoints, skill levels, and true mobile-hardware runtime testing.

Overall, the findings provide feasibility evidence for pose-based analysis of smartphone-recorded taekwondo kicks and for transparent, itemized feedback generation. The current system should be regarded as an offline, CPU-based desktop implementation evaluated in a feasibility study, rather than as a validated on-device mobile application or an established instructional intervention.

## Figures and Tables

**Table 1:** Acquisition protocol for smartphone-recorded videos.

Item	Description
Study setting	Routine university physical education classes
Participants	150 university students from five physical education classes
Class composition	Two first-year classes and three second-year classes
Skill background	First-year students had not yet learned the roundhouse kick; second-year students had completed instruction in the three target kicks.
Recorded kick types	Front kick, roundhouse kick, and axe kick
Attempts per participant	Two videos per assigned kick type
Protocol-level videos	790 smartphone-recorded videos
Recording device	Smartphone camera
Recording environment	Routine indoor teaching environment under available lighting
Additional sensors	None

**Table 2:** Dataset inclusion, exclusion, and current split summary.

Stage/Split	Participants	Front	Roundhouse	Axe	Total
Protocol expected	150	300	190	300	790
Retained active sequences	not encoded	288	190	287	765
Current train split	unavailable	245	159	244	648
Current validation split	unavailable	38	25	41	104
Release/example set	unavailable	5	6	2	13
Protocol minus retained	-	12	0	13	25

**Table 3:** Model architecture and training configuration.

Model	Input	Architecture	Dropout	Parameters	Training
BiLSTM	96 × 52	hidden = 128, bidirectional, layers = 2	0.2	582,403	Adam, lr = 1 × 10^−3^, batch = 8, epochs = 40
GCN	96 × 52	spatial skeleton GCN, d_model = 64, layers = 2	0.2	171,203	Adam, lr = 1 × 10^−3^, batch = 8, epochs = 40
Transformer	96 × 52	d_model = 128, layers = 2, heads = 4, FFN = 256	0.2	272,387	Adam, lr = 1 × 10^−3^, batch = 8, epochs = 40, label smoothing = 0.05

**Table 4:** Recognition backbone comparison under the current sample-level validation protocol. Values are reported as mean ± standard deviation across three random seeds. UNI-Argmax denotes uniform temporal resampling followed by standard argmax inference.

Model	Configuration	Accuracy (%)	Macro-F1 (%)	Weighted-F1 (%)	Balanced Acc. (%)	Seeds
LSTM	UNI-Argmax	75.3 ± 2.3	74.3 ± 2.3	75.3 ± 2.3	74.5 ± 2.3	3
LSTM	PA-Sweep	71.2 ± 1.0	69.9 ± 1.9	71.1 ± 1.1	70.3 ± 2.5	3
Transformer	PA-Sweep	67.3 ± 1.7	66.5 ± 1.3	67.9 ± 1.7	67.0 ± 1.1	3
Transformer	UNI-Argmax	62.2 ± 2.4	60.5 ± 2.1	61.8 ± 1.8	60.8 ± 1.9	3
GCN	PA-Sweep	51.0 ± 1.7	49.9 ± 1.9	50.4 ± 1.5	51.2 ± 2.9	3
GCN	UNI-Argmax	50.6 ± 1.1	50.1 ± 1.7	50.4 ± 2.0	51.3 ± 1.8	3

**Table 5:** Ablation of temporal normalization and inference strategy for the Transformer model. Values are reported as mean ± standard deviation across three random seeds. UNI-Argmax denotes uniform temporal resampling with standard argmax inference; PA-Sweep denotes class-conditioned phase-aligned resampling with sweep inference.

Configuration	Deployable	Accuracy (%)	Macro-F1 (%)	Balanced Acc. (%)	Interpretation
UNI-Argmax	Yes	62.2 ± 2.4	60.5 ± 2.1	60.8 ± 1.9	Controlled comparison
PA-SingleHyp.	Yes	65.4 ± 2.6	64.6 ± 2.1	65.2 ± 1.8	Controlled comparison
PA-Sweep	Yes	67.3 ± 1.7	66.5 ± 1.3	67.0 ± 1.1	Combined PA + sweep
PA-Oracle	No, analysis upper bound	69.2 ± 2.5	68.1 ± 2.0	68.5 ± 1.7	Controlled comparison

**Table 6:** Class-wise performance of the selected LSTM-UNI configuration.

Class	Precision (%)	Recall (%)	F1 (%)	Support
front	75.3 ± 3.2	72.8 ± 8.4	73.9 ± 5.8	38
roundhouse	66.6 ± 2.6	69.3 ± 4.6	67.9 ± 3.5	25
axe	80.8 ± 3.5	81.3 ± 3.8	81.0 ± 1.8	41

**Table 7:** Runtime performance on a video-based desktop prototype.

Stage	N	Mean (s/Video)	SD	Min	Max
Pose extraction	30	7.5099	4.5228	2.3224	20.2006
Feature preprocessing	30	0.0065	0.0045	0.0022	0.0234
Recognition inference	30	0.0030	0.0014	0.0022	0.0092
Rule-based scoring	30	0.0100	0.0040	0.0053	0.0242
Total excluding feedback rendering	30	7.5294	4.5303	2.3322	20.2514

**Table 8:** Human–AI agreement on the expert-annotated subset.

Metric	Result
Expert score reliability	ICC (A,3) = 0.915, 95% bootstrap CI [0.850, 0.949]
Class agreement vs. expert majority	83.3% (25/30), kappa = 0.750
Score rank correlation	Spearman rho = 0.627
Score linear correlation	Pearson r = 0.606
Score error	MAE = 0.601; MSE = 0.514
Bland–Altman	bias = +0.341; 95% LOA [−0.915, 1.598]

**Table 9:** Qualitative comparison with related Taekwondo assessment systems.

Study/System	Input Modality	Evaluated Movements	Explainability	Output
Hwang et al. [15]	Kinematic parameters and physical characteristics	Taekwondo Poomsae side kick	Feature-based interpretation	Performance classification
Fernando et al. [16]	MediaPipe-derived skeleton points	Taekwondo Poomsae movements	Limited rule/model transparency	Movement evaluation/accuracy result
Present study	Smartphone-recorded videos with MediaPipe pose	Front kick, roundhouse kick, and axe kick	Rule-based itemized scoring with deduction reasons	Kick recognition, 10-point score, deduction feedback, and visualized feedback video

## Data Availability

De-identified derived materials supporting the findings of this study, including pose-keypoint sequences, code, experiment manifests, and generated result files, are publicly available on Zenodo at https://doi.org/10.5281/zenodo.20390892. Raw videos are not publicly available due to privacy and ethical restrictions related to human participant recordings.

## Supplementary Materials

The following supporting information can be downloaded at: https://www.mdpi.com/article/10.3390/s26144499/s1, Videos S1–S3: Three anonymized supplementary video demonstrating the generated visual feedback output, including the skeleton overlay, recognition result, scoring information, and deduction-based feedback. Potentially identifying facial information was obscured before submission.
